# Pharmacological evaluation of the semi-purified fractions from the soft coral *Eunicella singularis* and isolation of pure compounds

**DOI:** 10.1186/s40199-014-0064-7

**Published:** 2014-09-10

**Authors:** Monia Deghrigue, Carmen Festa, Lotfi Ghribi, Maria Valeria D’auria, Simona de Marino, Hichem Ben Jannet, Rafik Ben Said, Abderrahman Bouraoui

**Affiliations:** Laboratoire de développement chimique, galénique et pharmacologique des médicaments (LR12ES09). Equipe de Pharmacologie marine, Faculté de pharmacie de Monastir, Université de Monastir, Monastir, Tunisie; Department of Pharmacy, University of Naples “Federico II”, via D. Montesano 49, I- 80131 Napoli, Italy; Laboratoire de chimie hétérocyclique, produits naturels et réactivité. Equipe de chimie médicinale et produits naturels (LR11ES39), Faculté des sciences de Monastir, Université de Monastir, Monastir, Tunisie; Institut National des Sciences et Technologie de la Mer (INSTM), Salambo Tunis, Tunisie

**Keywords:** *Eunicella singularis*, Anti-inflammatory activity, Gastroprotective effect, Marine natural product, Diterpenoid, Sterols

## Abstract

**Background:**

Gorgonians of the genus *Eunicella* are known for possessing a wide range of pharmacological activities such as antiproliferative and antibacterial effect. The aim of this study was to evaluate the anti-inflammatory and gastroprotective effect of the organic extract and its semi-purified fractions from the white gorgonian *Eunicella singularis* and the isolation and identification of pure compound(s) from the more effective fraction.

**Methods:**

Anti-inflammatory activity was evaluated, using the carrageenan-induced rat paw edema test and in comparison to the reference drug Acetylsalicylate of Lysine. The gastroprotective activity was determined using HCl/EtOH induced gastric ulcers in rats. The purification of compound(s) from the more effective fraction was done by two chromatographic methods (HPLC and MPLC). The structure elucidation was determined by extensive spectroscopic analysis (^1^H and ^13^C NMR, COSY, HMBC, HMQC and NOESY) and by comparison with data reported in the literature.

**Results:**

The evaluation of the anti-inflammatory activity of different fractions from *Eunicella singularis* showed in a dependent dose manner an important anti-inflammatory activity of the ethanol fraction, the percentage of inhibition of edema, 3 h after carrageenan injection was 66.12%, more effective than the reference drug (56.32%). In addition, this ethanolic fraction showed an interesting gastroprotective effect compared to the reference drugs, ranitidine and omeprazol. The percentage of inhibition of gastric ulcer induced by HCl/ethanol in rats was 70.27%. The percentage of the reference drugs (ranitidine and omeprazol) were 65 and 87.53%, respectively. The purification and structure elucidation of compound(s) from this ethanolic fraction were leading to the isolation of five sterols: cholesterol (5α-cholest-5-en-3β-ol) **(1)**; ergosterol (ergosta-5,22-dien-3β-ol) **(2)**; stigmasterol (24-ethylcholesta-5,22-dien-3b-ol) **(3)**; 5α,8α-epidioxyergosta 6,22-dien-3β-ol **(4)** and 3β-hydroxy-5α,8α-epidioxyergosta-6-ene **(5);** and one diterpenoid: palmonine D **(6).**

**Conclusion:**

Based on data presented here, we concluded that diterpenoids and sterols detected in the ethanolic fraction can be responsible for its pharmacological activity.

**Electronic supplementary material:**

The online version of this article (doi:10.1186/s40199-014-0064-7) contains supplementary material, which is available to authorized users.

## Background

Nature has developed an enormous diversity during several billion years of evolution. The Mediterranean Area represents one of the world’s major centers of animal diversity; with around 20 gorgonian species, four belong to the genus *Eunicella*: *E. verrucosa*, *E. filiformis*, *E. cavolini* and *E. singularis* [1].

Although natural compounds have been replaced by synthetic chemistry as the main source of new drugs, marine invertebrates remain an unequalled source of biochemical diversity. The studies on gorgonian have great importance in the research of marine resources of active compounds mainly by the pharmaceutical industry or for other uses. In fact, the gorgonians (Anthozoa, Gorgonacea) are known for possessing a wide range of pharmacologic and health promoting properties including antibacterial [2], antiviral [3], antiplasmodial [4], antifouling [5], antiproliferative [6], cytotoxic [7] and insecticidal [8] effects. The gorgonian of the genus *Eunicella* has been demonstrated to contain a wide variety of natural products as steroids and diterpenes [9,10]. These compounds posses anticancer, gastroprotective and anti-inflammatory activities [11]. For many years, our marine pharmacological group in Tunisia has been involved in an accurate research program on gorgonian constituents in order to define both their chemical composition and their biological activities. On the other hand, the use of non-steroidal anti-inflammatory drugs (NSAID) for the treatment of inflammatory diseases is associated with adverse effects as peptic ulcer [12]. Therefore, the research of potent anti-inflammatory drugs from natural sources and with fewer side effects had become necessary. This study has yielded the anti-inflammatory and gastroprotective effects of the organic extract and its semi-purified fractions of the white gorgonian *Eunicella singularis* (Esper, 1791). The structure elucidation of the isolated compounds from the active fraction was done by 1D and 2D NMR experiments and by comparison with literature data.

## Methods

### General procedures

HPLC was performed using a Waters model 510 pump equipped with Waters Rheodine injector and a differential refractometer, model 401. Medium pressure liquid chromatography (MPLC) was performed on a Buchi apparatus using a silica gel (230–400 mesh) column.

NMR spectra were obtained on Varian Inova 400 and Varian Inova 500 NMR spectrometers (^1^H at 400 and 500 MHz, ^13^C at 100 and 125 MHz, respectively) equipped with a Sun hardware, δ (ppm), *J* in hertz, and spectra referred to CD_3_Cl_3_ (δH=7.27; δC= 70.0 ) as internal standard. High-resolution ESIMS spectra were performed with a Micromass QTOF Micro mass spectrometer. All reagents were commercially obtained (Aldrich, Fluka) at the highest commercial quality and used without further purification except where noted. All reactions were monitored by TLC on silica gel plates (Macherey–Nagel). Carrageenan (BDH Chemicals Ltd Poole England), Acetylsalicylate of Lysine (ASL) were purchased from Sigma Chemical (Berlin, Germany). Ranitidine was obtained from Medis (Tunis, Tunisia), omeprazole was obtained from AstraZeneca (Monts).

### Collection and extraction

*E. singularis* was collected from the Mediterranean Sea in various areas of the coastal region of Tabarka (Tunisia), in June 2010, at a depth between 20 and 30 m. Identification of specimens was carried out in the National Institute of Marine Sciences and Technologies (Salamboo, Tunisia) where a voucher specimen of *E. singularis* was deposited under the following reference 1132. After maceration of 600 g of the powdered material with methanol and dichloromethane (1:1, v/v) for 48h three times, the organic extract (40 g) was purified, using C18 cartridges (Sep-pack, Supelco), by gradient elution with different organic solvents in the order of decreased polarity: ethanol, acetone and methanol/CH_2_Cl_2_ (1:1) to give three semi-purified fractions: ethanol (F-EtOH), acetone (F-Ac) and methanol/CH_2_Cl_2_ (F-MeOH/CH_2_Cl_2_) fractions. Organic solvents were removed from recuperated fractions using rotating evaporator at 40°C.

### Purification, isolation and structure elucidation

**Figure 1:**
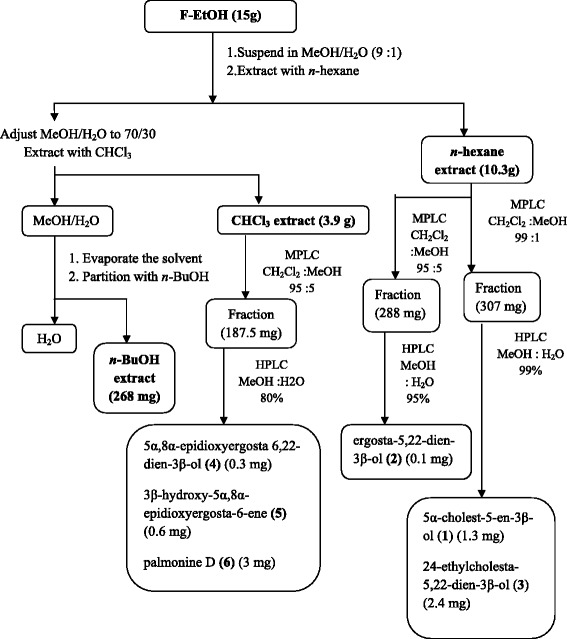
**Purification and separation process of the isolation of six compounds from the ethanolic fraction from** ***E. singularis.***

F-EtOH (15 g) was fractionated according to the Kupchan partitioning procedure [13] as follow: the ethanolic fraction was dissolved in a mixture of MeOH/H_2_O containing 10% H_2_O and partitioned against *n*-hexane to give 10.3 g of the crude extract. The water content (% v/v) of the MeOH extract was adjusted to 30% and partitioned against CHCl_3_ to give 3.9 g of the crude extract. The aqueous phase was concentrated to remove MeOH and then extracted with *n*-BuOH (268 mg of crude extract) (Figure 1). The *n*-hexane extract (5 g) was fractioned by silica gel MPLC using a solvent gradient system from CH_2_Cl_2_ to MeOH. Fraction eluted with CH_2_Cl_2_: MeOH 99:1 (307 mg) was purified by HPLC on a Nucleodur 100–5 C18 (5 μm; 10 mm i.d. × 250 mm) with 99% MeOH: H_2_O as eluent (flow rate 3mL/min) to give 1.3 mg of 5α-cholest-5-en-3β-ol (**1**) (t_R_=55 min) and 2.4 mg of 24-ethylcholesta-5, 22-dien-3β-ol (**3**) (t_R_=83min) (Figure 1).

Fraction eluted with CH_2_Cl_2_:MeOH 95:5 (288 mg) was purified by HPLC on a Nucleodur 100–5 C18 (5 μ, 4.6 mm i.d. × 250 mm) with 95% MeOH:H_2_O as eluent (flow rate 1 mL/min) to give 0.1 mg of ergosta-5,22-dien-3β-ol (**2**) (t_R_=74 min) (Figure 1).

The CHCl_3_ extract (3.9 g) was chromatographed by silica gel MPLC using a solvent gradient system from CH_2_Cl_2_ to CH_2_Cl_2_: MeOH 1:1.

Fraction eluted with CH_2_Cl_2_: MeOH 95:5 (187.5 mg) was further purified by HPLC on a Nucleodur 100–5 C18 (5 μ, 4.6 mm i.d. × 250 mm) with 80% MeOH:H_2_O as eluent (flow rate 1 mL/min) to give 0.3 mg of 5α,8α-epidioxyergosta 6,22-dien-3β-ol (**4**) (t_R_=29 min), and 0.6 mg of 3β-hydroxy-5α,8α-epidioxyergosta-6-ene (**5**) (t_R_=2 min) and 3 mg of palmonine D (**6**) (t_R_=3 min) (Figure 1).

The purities of compounds were determined to be greater than 95% by HPLC and NMR. Furthermore, HPLC chromatograms and spectroscopic data of each compound were reported (Additional file 1).

### Pharmacological evaluation

#### Animals

Wistar rats of either sex, weighing 150–200 g were obtained from Pasteur Institute (Tunis, Tunisia). Housing conditions and *in vivo* experiments were approved according to the guidelines established by the European Union on Animal Care (CCE Council 86/609).

#### Carrageenan-Induced Rat Paw Edema

The anti-inflammatory activity of the organic extract and its semi-purified fractions on carrageenan-induced paw edema was determined according to Winter et al. [14]. The animals were divided into eleven groups of six rats each. The control group received an intraperitoneal (i.p.) dose of saline solution (NaCl 9g/L, 2.5 mL/kg), the reference group received Acetylsalicylate of Lysine (ASL) (300 mg/kg, i.p.), and the test groups received the organic extract of *E. singularis* (50, 100 and 200 mg/kg, i.p.) and its semi-purified fractions F-EtOH, F-Ac and F-MeOH/CH_2_Cl_2_ (25 and 50 mg/kg, i.p.). After 30 min, 0.05mL of a 1% carrageenan suspension was injected into the left hind paw. The paw volume up to the tibiotarsal articulation was measured using a plethysmometer (model 7150, Ugo Basile, Italy). The measures were determined at 0 h (V_0_) (before carrageenan injection) and 1, 3 and 5 h later (V_T_) (after carrageenan injection). Paw swelling was determined for each rat and the difference between V_T_ and V_0_ was taken as the edema value. The percentages of inhibition were calculated according to the following formula:

$$ \%\ \mathrm{inhibition} = \left[{\left({\mathrm{V}}_{\mathrm{T}}-{\mathrm{V}}_0\right)}_{\mathrm{control}}\hbox{--}\ {\left({\mathrm{V}}_{\mathrm{T}}-{V}_0\right)}_{\mathrm{treated}\ \mathrm{group}}/\ {\left({\mathrm{V}}_{\mathrm{T}}-{\mathrm{V}}_0\right)}_{\mathrm{control}}\right] \times 100 $$

#### Gastric lesions induced by HCl/ethanol

The gastroprotective activity of the organic extract of *E. singularis* and its semi-purified fractions F-EtOH, F-Ac and F-MeOH/CH_2_Cl_2_ was studied in 150 mM HCl/EtOH induced gastric ulcer [15]. Rats were divided into fifteen groups, fasted for 24 h prior receiving an intraperitoneal doses of vehicle (NaCl 9g/L, 2.5 mL/kg) for the control group, organic extract (50, 100 and 200 mg/kg, i.p.) and its semi-purified fractions F-EtOH, F-Ac and F-MeOH/CH_2_Cl_2_ (5, 10 and 25 mg/kg, i.p.) for the twelve test groups. Two other groups received ranitidine (60 mg/kg, i.p.) and omeprazole (30 mg/kg, i.p.) as reference drugs. After 30 min, all groups were orally treated with 1mL of 150 mM HCl/EtOH solution for gastric ulcer induction. Animals were killed 1 h after the administration of ulcerogenic agent, their stomachs were excised and opened along the great curvature, washed and stretched on cork plates. The surface was examined for the presence of lesions and the extent of the lesions was measured. The summative length of the lesions along the stomach was recorded (mm) as lesion index.

#### Statistical analysis

Data are presented as the mean±standard error (s.e.m). Statistical analysis was performed using Student’s t-test. The significance of difference was considered to include values of P<0.05.

## Results and discussion

The current study was carried out to determine the in *vivo* anti-inflammatory and gastroprotective activities of the organic extract of *E. singularis* and its semi-purified fractions. The chemical composition of the more effective fraction was determined by both 1D and 2D NMR experiments.

**Figure 2:**
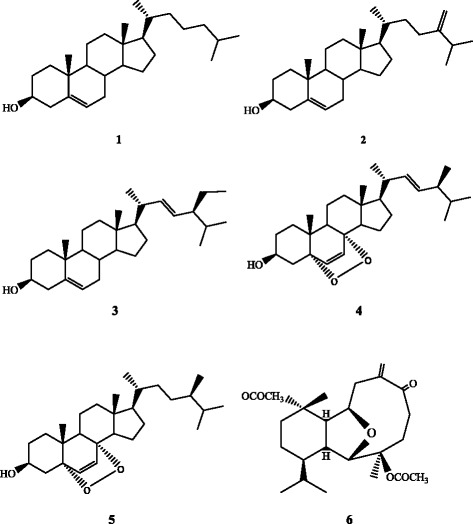
**Chemical structures of six compounds isolated from the white gorgonian** ***E. singularis*** **.** Five sterols **(1-5)**: 5α-cholest-5-en-3β-ol **(1)**; ergosta-5,22-dien-3β-ol **(2)**; 24- ethylcholesta-5,22-dien-3b-ol **(3)**; 5α,8α-epidioxyergosta 6,22-dien-3β-ol **(4)** and 3β-hydroxy- 5α,8α-epidioxyergosta-6-ene **(5);** and one diterpenoid: palmonine D **(6)**.

As shown in Figure 2, 1D and 2D NMR analysis of the ethanolic fraction (F-EtOH) from the gorgonian *E. singularis* resulted in the identification of six compounds.

Compound **1** was isolated as colorless powder. The molecular formula was determined to be C_27_H_46_O. Analysis of ^1^H and ^13^C NMR data evidenced a Δ^5^ dihydroxy-steroid structure with a saturated C8 cholestane side chain. Comparison with literature data allowed assigning the 5α-cholest-5-ene-3β-ol (cholesterol) structure [16].

Compound **2** has a molecular formula of C_28_H_46_O as determined by HRESIMS. Compound **2** was identified as ergosta-5,22-dien-3β-ol (ergosterol) [17] (Figure 2). This compound was also isolated from the soft coral *Tubastraea coccinea* and *T. tagusensis* [17].

Compound **3** was isolated as colorless powder. The molecular formula was determined to be C_28_H_46_O_._ Its identity was determined by 1D and 2D NMR data as 24-ethylcholesta-5,22-dien-3b-ol (stigmasterol) earlier isolated from the halophyte *Salicornia herbacea* [18] and then reported also from the plant *Aglaia eximia* [19].

Compound **4** was isolated as white powder. The molecular formula was determined to be C_28_H_44_O_3_ by HRESIMS data. NMR data disclosed the 5α,8α-epidioxyergosta 6,22-dien-3β-ol structure. This compound was also isolated from the fungus *Sporothrix schenckii* [20] and the fungus *Cryptoporus volvatus* [21].

Compound **5** has a molecular formula of C_28_H_46_O_3_ as determined by HRESIMS. ^1^H and ^13^C NMR indicated that compound **5** is the Δ^22^ derivative of compound **4**. Therefore compound **5** was identified as 3β-hydroxy-5α,8α-epidioxyergosta-6-ene [21].

Compound **6** was isolated as colorless oil. The molecular formula was determined to be C_24_H_36_O_6_ by HRESIMS. The analysis of ^1^H NMR spectrum clearly revealed an eunicellin diterpenoid structure. The ^13^C NMR spectrum revealed 24 carbon signals. ^1^H- and ^13^CNMR assignments were carried out with the aid of the detailed 2D analyses (COSY, HMQC, NOESY, and HMBC) and the resulting NMR evidence revealed **6** to be defined as palmonine D [22]. Three sterols named 5α, 8α-epidioxysterols, pregnanes and 9,11-secosterols were separated from *E. cavolini*, another specie of the genus *Eunicella* [10,11]. The five sterols identified in our study were isolated for the first time from *E. singularis* and were not yet found with another specie from this genus.

Palmonine D is also purified from *E. labiata*, another specie of the genus *Eunicella* [22]. Other researchers reported the isolation of five diterpenoids from the gorgonian *E. labiata,* labiatamide A, labiatamide B, labiatin A, labiatin B and labiatin C [23] but these compounds were not yet separated from *E. singularis*. Massileunicellin A, was obtained from *E. cavolini* [24] but also was not identified from *E.singularis*.

**Figure 3:**
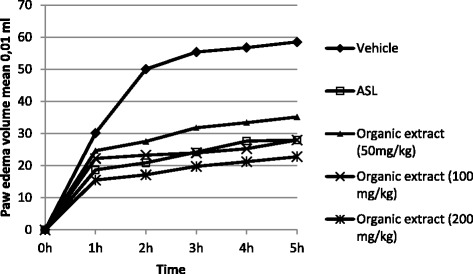
**Effect of intraperitoneal administration of the organic extract from** ***E. singularis*** **on the carrageenan-induced rat paw edema.** Values are mean ± SEM.

**Figure 4:**
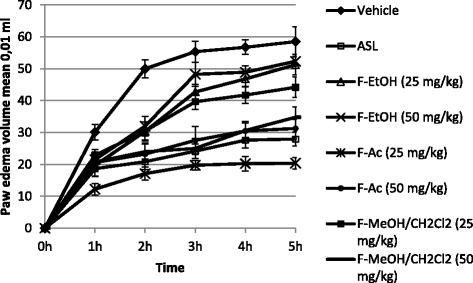
**Effect of intraperitoneal administration of semi-purified fractions (F-Ac, F-EtOH and F-MeOH/CH2Cl2 from** ***E. singularis*** **on the carrageenan-induced rat paw edema.** Values are mean ± SEM.

The results reported in Figures 3 and 4 showed the anti-inflammatory effects of organic extract and its semi-purified fractions from *E. singularis* administered intraperitoneally.

*E. singularis* organic extract presented a dose- related, statistically significant anti-inflammatory activity on carrageenan induced paw edema which was comparable with the reference drug, Acetylsalicylate of Lysine (ASL), a potent inhibitor of cyclooxygenase-2. The percent inhibition of edema at dose levels of 50, 100 and 200 mg/kg were 42.61%, 56.47% and 64.31% (at 3 h), respectively (Table 1). The semi-purified fractions (F-Ac, F-EtOH, F-MeOH/CH_2_Cl_2_) were assessed for anti-inflammatory effect at 25 and 50 mg/kg. A dose-related anti-inflammatory property was observed for the three fractions with highly significant activity of F-EtOH at dose 50 mg/kg with a percentage of inhibition of 66.12% at 3 h. While F-Ac and F-MeOH/CH_2_Cl_2_ at a dose of 50 mg/kg reduced edema with a percentage of 50.22 and 54.78% (at 3 h), respectively. ASL as a reference standard drug inhibited the edema formation due to carrageenan to an extent of 56.32% (at 3 h) at the dose of 300 mg/kg. The development of edema induced by carrageenan corresponds to the events in the acute phase of inflammation mediated by histamine, bradykinin and prostaglandins produced under an effect of cyclooxygenase-2 (COX-2) [25]. This enzyme is an inducible cyclooxygenase which boosts the inflammatory response by COX-2 mediated prostaglandin E_2_ (PGE_2_) [26]. Hence, it is probably that the organic extract and its semi-purified fractions from the gorgonian *E. singularis* reduced inflammation by blocking the cyclooxygenase2 (COX-2). Morever, several studies reported that steroids isolated from other species of the genus *Eunicella* have anti-inflammatory activity such as secosteroids [11]. The high anti-inflammatory activity of the fraction F-EtOH can be related with the presence of its main steroid constituents. The chemical analysis of this fraction (F-EtOH) revealed the presence of five sterols: 5α-cholest-5-en-3β-ol (cholesterol) **(1)**; ergosta-5,22-dien-3β-ol (ergosterol) **(2)**; 24-ethylcholesta-5,22-dien-3b-ol (stigmasterol) **(3)**; 5α,8α-epidioxyergosta 6,22-dien-3β-ol **(4)** and 3β-hydroxy-5α,8α-epidioxyergosta-6-ene **(5)**.

**Table 1:** **Anti-inflammatory effect of the intraperitoneal administration of** ***E. singularis*** **organic extract and its semi-purified fractions (F-EtOH, F-Ac and F-MeOH/CH**_**2**_ **Cl**_**2**_ **) and of reference drug (Acetylsalicylate of Lysine; ASL) in carrageenan-induced rat paw edema test**

**Treatment**	**Dose (mg/kg)**	**Edema (10** ^**−2**^ **ml) (mean±s. e. m)**			**Edema inhibition (%)**		
		**1 h**	**3 h**	**5 h**	**1 h**	**3 h**	**5 h**
Vehicle	-	30.12±2.38	55.35±3.3	58.5±4.69	-	-	-
Organic extract	50	24.62±2.8	31.76±3.6**	35.14±4.1*	18.26	42.61	39.92
	100	22.22±2.9	23.94±4.1**	27.95±1.5**	32.84	56.47	52.21
	200	15.5±3.2*	19.75±2.4**	22.75±3.6**	48.53	64.31	61.11
F-EtOH	25	19.73±1.6	42.74±2.7	51.26±2.6	34.49	22.78	12.36
	50	12.25±1.5**	18.75±2**	20.37±2.4**	59.32	66.12	65.17
F-Ac	25	21.55±2.6	48.24±2.4	52.33±3.5	28.42	12.84	10.53
	50	20.63±2.4	27.55±3.2**	31.27±2.7*	31.48	50.22	46.54
F-MeOH/CH_2_Cl_2_	25	22.85±3.2	39.67±2.6	44.2±4.8	24.12	28.32	24.43
	50	20.82±2.4	25.02±2.9**	34.79±3.4*	30.86	54.78	40.52
ASL	300	19.66±3.4	24.17±2.7**	27.95±3.1**	34.71	56.32	52.22

Values are expressed as mean±SEM; n=6 animals. *P<0.01, **P<0.001.

The gastroprotective effect of *E. singularis* organic extract and its semi-purified fractions against HCl/EtOH induced gastric damage in rats is shown in (Table 2) and the results are comparable to that of the reference drugs ranitidine, histamine H_2_ receptor antagonist, and omeprazole, a proton pump inhibitor [27]. Oral administration of HCl/EtOH produced gastric mucosal damage with severe hemorrhage with lesion index of 78.5 mm in the untreated group. Treatment of rats by organic extract of *E. singularis* produced a significant decrease in gastric hemorrhage and the lesion index was inhibited by 60, 68 and 75% at doses of 50, 100 and 200 mg/kg, respectively. The semi-purified fractions were assessed for gastroprotective activity at 5, 10 and 25 mg/kg. A dose-related gastroprotective effect was observed for the fractions F-EtOH and F-MeOH/CH_2_Cl_2_ with highly significant activity for the ethanolic fraction (F-EtOH) at 25 mg/kg. The lesion index was inhibited by 44, 52 and 70% at doses of 5, 10 and 25 mg/kg, respectively; while in the ranitidine treated animals (60 mg/kg) the inhibition was 65%. F-Ac failed to protect stomach tissues from mucosal damage. The two classical ulcer drugs ranitidine and omeprazole showed a significant activity with a percentage of inhibition of gastric lesions of 65 and 87%, respectively. Some reports on the gastroprotective effect of diterpenes belonging to different structural skeletons are published [28]. In addition, another studies demonstrated that several terpenes or their derivatives posses gastroprotective activity in different models of induced gastric lesions in animals [29]. This gastroprotective effect seems to be related with an increase of the defensive mechanisms of the stomach, such as prostaglandin synthesis and mucus production [30]. Therefore, the involvement of diterpenoids palmonine D **6** isolated from the active fraction F-EtOH is hypothesized and can be responsible for its high activity.

**Table 2:** **Effect of** ***E. singularis*** **organic extract and its semi-purified fractions (F-EtOH, F-Ac, F-MeOH/CH**_**2**_ **Cl**_**2**_ **), and of reference drugs (ranitidine and omeprazol) on gastric ulcer induced by HCl/ethanol in rats**

**Treatment**	**Dose (mg/kg)**	**Ulcer index (mm)**	**Inhibition (%)**
Vehicle	-	78.5±3,49	-
Organic extract	50	31.52±2,42**	59.84
	100	24.46±2,48**	68.84
	200	19.49±3,48**	75.16
F-EtOH	5	43.36±1,52*	44.76
	10	37.20±3,05**	52.61
	25	23.33±1**	70.27
F-Ac	25	61.97±0,7	21.05
F-MeOH/CH_2_Cl_2_	5	66.59±2,33	15.17
	10	58.78±2,82	25.11
	25	29.17±4,04**	62.84
Ranitidine	60	27.47±2.3**	65
Omeprazol	30	9.78±0,81*	87.53

Values are expressed as mean±SEM; n=6 animals. *P<0.01, **P<0.001.

Furthermore, various phenolic compounds (alkaloids, glycosides, and saponins) detected in *E. singularis* organic extract and fractions [6] may be responsible for its activity. Several studies reported that alkaloids have anti-inflammatory and gastroprotective effects [31]. Also, Glycosides, terpenoids and steroids detected in our samples are known to have anti-inflammatory and gastroprotective properties [10,32] The synergic effect of different compounds of *E. singularis* ethanolic fraction may be responsible for its higher anti-inflammatory and gastroprotective activities. Furthermore, the high free radical scavenging activity of F-EtOH in the DPPH test [6] suggests that the antioxidant activity may be one of the mechanisms of its gastroprotective and anti-inflammatory properties, because both ulcerous and inflammatory processes are related to an increase of free radicals [33].

## Conclusion

In conclusion, the obtained results demonstrated that the ethanolic fraction of *E. singularis* had the highest activity in the two tests (anti-inflammatory and gastroprotective). The structure elucidation of compounds isolated from this fraction revealed the presence of five sterols and a eunicellan-based diterpenoid which may be responsible for its activity.

## Additional file

Additional file 1:
**HPLC chromatograms and spectroscopic data of six compounds isolated from the white gorgonian** ***E. singularis.***

## References

[1] Gori A, Bramanti L, Lopez-Gonzalez P, Thoma JN, Gili GM, Grinyo J, Uceira V, Rossi S (2012). Characterization of the zooxanthellate and azooxanthellate morphotypes of the Mediterranean gorgonian *Eunicella singularis*. Mar Biol.

[2] McEnroe FJ, Fenical W (1978). Structures and synthesis of some new antibacterial sesquiterpenoids from the gorgonian coral *Pseudopterogorgia rigida*. Tetrahedron.

[3] Groweiss A, Look S, Fenical W (1988). Solenolides, new antiinflammatory and antiviral diterpenoids from a marine octocoral of the genus *Solenopodium*. J Org Chem.

[4] Wei X, Rodriguez AD, Baran P, Raptis RG, Sanchez JA, Ortega-Barria E, Gonzalez J (2004). Antiplasmodial cembradiene diterpenoids from a Southwestern Caribbean gorgonian octocoral of the genus *Eunicea*. Tetrahedron.

[5] Qi SH, Zhang S, Qian PY, Xiao ZH, Li MY (2006). Ten new antifouling briarane diterpenoids from the South China Sea gorgonian *Junceella juncea*. Tetrahedron.

[6] Deghrigue M, Dellai A, Bouraoui A (2013). In vitro antiproliferative and antioxidant activities of the organic extract and its semi-purified fractions from the Mediterranean gorgonian *Eunicella singularis*. Int J Pharm Pharm Sci.

[7] Sheu JH, Sung PJ, Cheng MC, Liu HY, Fang LS, Duh CY, Chiang MY (1998). Novel Cytotoxic Diterpenes, Excavatolides A-E, Isolated from the Formosan Gorgonian *Briareum excavatum*. J Nat Prod.

[8] Grode SH, James TR, Cardellina JH, Onan KD (1983). Molecular structures of the briantheins, new insecticidal diterpenes from *Briareum polyanthes*. J Org Chem.

[9] Berrue F, Kerr RG (2009). Diterpenes from gorgonian corals. Nat Prod Rep.

[10] Ioannou E, Abdel-Razik AF, Alexi X, Vagias C, Alexis MN, Roussis V (2008). Pregnanes with antiproliferative activity from the gorgonian *Eunicella cavolini*. Tetrahedron.

[11] Ioannou E, Abdel-Razik AF, Alexi X, Vagias C, Alexis MN, Roussis V (2009). 9,11-Secosterols with antiproliferative activity from the gorgonian *Eunicella cavolini*. Bioorg Med Chem.

[12] Hossain H, Al-Mansur A, Akter S, Sara U, Ahmed MR, Jahangir AA (2014). Evaluation of anti-inflammatory activity and total tannin content from the leaves of *Bacopa monnieri* (Linn.). IJPSR.

[13] Kupchan SM, Britton RW, Ziegler MF, Sigel CW (1973). Bruceantin, a new potent antileukemic simaroubolide from *Brucea antidysenterica*. J Org Chem.

[14] Winter CA, Risley EA, Nuss GW (1962). Carrageenan induced edema hind paw of the rat as an easy for anti-inflammatory drugs. Proc Soc Exp Biol Med.

[15] Mizui T, Doteuchi M (1983). Effect of polyamines on acidified ethanol-induced gastric lesions in rats. Japanese J Pharmacology.

[16] Acimovic J, Rozman D (2013). Steroidal triterpenes of cholesterol synthesis. Molecules.

[17] Lages BG, Fleury BG, Hovell AMC, Rezende CM, Pinto AC, Creed JC (2012). Proximity to competitors changes secondary metabolites of non-indigenous cup corals, *Tubastraea* spp., in the southwest Atlantic. Mar Biol.

[18] Wang X, Zhang M, Zhao Y, Wang H, Liu T, Xin Z (2013). Pentadecyl ferulate, a potent antioxidant and antiproliferative agent from the halophyte *Salicornia herbacea*. Food Chem.

[19] Harneti D, Supriadin A, Ulfah M, Safari A, Supratman U, Awang K, Hayashi H (2014). Cytotoxic constituents from the bark of *Aglaia eximia* (Meliaceae). Phytochem Lett.

[20] Sgarbi DBG, da Silva AJR, Carlos IZ, Silva CL, Angluster J, Alviano CS (1997). Isolation of ergosterol peroxide and its reversion to ergosterol in the pathogenic fungus *Sporothrix schenckii*. Mycopathologia.

[21] Wei-Guang M, Xing-Cong L, De-Zu W, Chong-Ren Y (1994). Ergosterol peroxides from *Cryptoporus volvatus*. Acta Bot Yunnanica.

[22] Ortega MJ, Zubia E, Salva J (1997). A new cladiellane diterpenoid from *Eunicella labiata*. J Nat Prod.

[23] Roussis V, Fenical W, Vagias C, Kornprobst JM, Miralles J (1996). Labiatamides A, B, and other eunicellan diterpenoids from the Senegalese gorgonian *Eunicella labiata*. Tetrahedron.

[24] Hanson JR (2001). Diterpenoids. Nat Prod Rep.

[25] Borgi W, Ghedira K, Chouchane N (2007). Antiinflammatory and analgesic activities of Zizyphus lotus root barks. Fitoterapia.

[26] Inoue H, Ohshima H, Kono H, Yamanaka M, Kubota T, Aihara M, Hiroi T, Yago N, Ishida H (1997). Supressive effects of tranilast on the expression of inducible cyclooxygenase (COX-2) in interleukin-1-β-stimulated fibroblasts. Biochem Pharmacol.

[27] Ishihara M, Ito M (2002). Influence of aging on gastric ulcer healing activities of cimetidine and omeprazole. Eur J Pharmacol.

[28] Schmeda-Hirschmann G, Astudillo L, Rodriguez J, Theoduloz C, Yanez T (2005). Gastroprotective effect of the Mapuche crude drug Araucaria araucana resin and its main constituents. J Ethnopharmacol.

[29] Farina C, Pinza M, Pifferi G (1998). Synthesis and anti-ulcer activity of new derivatives of glycyrrhetic, oleanolic and ursolic acids. Il Farmaco.

[30] Hiruma-Lima CA, Gracioso JS, Toma W, Paula ACB, Almeida ABA, Brasil DD, Muller AH, Souza-Brito AR (2000). Evaluation of the gastroprotective activity of cordatin, a diterpene isolated from Aparisthmium cordatum (Euphorbiaceae). Biol Pharm Bull.

[31] Moulin M, Coquerel A (2002). Pharmacologie, connaissance et pratique.

[32] Radjasa OK, Vaske YM, Navarro G, Vervoort HC, Tenney K, Linington RG, Crews P (2011). Highlights of marine invertebrate-derived biosynthetic products: their biomedical potential and possible production by microbial associants. Bioorg Med Chem.

[33] Pedernera AM, Guardia T, Guardia Calderon C, Rotelli AE, de la Rocha NE, Di Genaro S, Pelzer LE (2006). Anti-ulcerogenic and anti-inflammatory activity of the methanolic extract of *Larrea divaricata* Cav. In rats. J Ethnopharmacol.

