# Supplementary material for: Pharmacological evaluation of the semi-purified fractions from the soft coral Eunicella singularis and isolation of pure compounds
Source: Daru. 2014 Sep 10;22(1):64. doi: 10.1186/s40199-014-0064-7 (PMC4172910; doi:10.1186/s40199-014-0064-7)
Supplement: Additional file 1: — HPLC chromatograms and spectroscopic data of six compounds isolated from the white gorgonian E. singularis. [file 40199_2014_64_MOESM1_ESM.docx]

**Additional files for the editors**

**Figure 1 HPLC of *Eunicella* compound 1 (5α-cholest-5-en-3β-ol (1); Compound 1 = pick 22)**

**Figure 2 ^1^H NMR of *Eunicella* compound 1 (5α-cholest-5-en-3β-ol) in CDCl_3_ (500 MHz)**

**Figure 3 COSY of *Eunicella* compound 1 (5α-cholest-5-en-3β-ol) in CDCl_3_ (500 MHz)**

**Figure 4 NOESY of *Eunicella* compound 1 (5α-cholest-5-en-3β-ol) in CDCl_3_ (500 MHz, 250 ms)**

**Figure 5 HPLC of *Eunicella* compound 2 (ergosta-5,22-dien-3β-ol) and 3 (24-ethylcholesta-5,22-dien-3b-ol); Compound 2 = pick 19; Compound 3 = pick 20**

**Figure 6 ^1^H NMR of *Eunicella* compound 2 (ergosta-5,22-dien-3β-ol) in CDCl_3_ (500 MHz)**

**Figure 7 ^1^H NMR of *Eunicella* compound 3 (24-ethylcholesta-5,22-dien-3b-ol) in CDCl_3_ (500 MHz)**

**Figure 8 HPLC of Eunicella compounds 4 (5α,8α-epidioxyergosta 6,22-dien-3β-ol), 5 (3β-hydroxy-5α,8α-epidioxyergosta-6-ene) and 6 (palmonine D); Compound 4= pick 21; Compound 5= pick 3; Compound 6= pick 4**

**Figure 9 ^1^H NMR of *Eunicella* compound 4 (5α,8α-epidioxyergosta 6,22-dien-3β-ol) in CDCl_3_ (400 MHz)**

**Figure 10 ^1^H NMR of *Eunicella* compound 5 (3β-hydroxy-5α,8α-epidioxyergosta-6-ene) in CDCl_3_ (500 MHz)**

**Figure 11 ^1^H NMR of *Eunicella* compound 6 (palmonine D) in CDCl_3_ (500 MHz)**

**Figure 12 COSY of *Eunicella* compound 6 (palmonine D) in CDCl_3_ (500 MHz)**

**Figure 13 HMBC of *Eunicella* compound 6 (palmonine D) in CDCl_3_ (500 MHz)**

**Figure 14 NOESY of *Eunicella* compound 6 (palmonine D) in CDCl_3_ (500 MHz, 250 ms)**
